# Lipid Identification and Transcriptional Analysis of Controlling Enzymes in Bovine Ovarian Follicle

**DOI:** 10.3390/ijms19103261

**Published:** 2018-10-20

**Authors:** Priscila Silvana Bertevello, Ana-Paula Teixeira-Gomes, Alexandre Seyer, Anaïs Vitorino Carvalho, Valérie Labas, Marie-Claire Blache, Charles Banliat, Luiz Augusto Vieira Cordeiro, Veronique Duranthon, Pascal Papillier, Virginie Maillard, Sebastien Elis, Svetlana Uzbekova

**Affiliations:** 1 UMR PRC, INRA 85, CNRS 7247, Université de Tours, IFCE, 37380 Nouzilly, France; priscila.bertevello@inra.fr (P.S.B.); anais.carvalho@hotmail.fr (A.V.C.); valerie.labas@inra.fr (V.L.); marie-claire.blache@inra.fr (M.-C.B.); Charles.Banliat@inra.fr (C.B.); Luiz-Augusto.Viera-Cordeiro@inra.fr (L.A.V.C.); pascal.papillier@inra.fr (P.P.); virginie.maillard@inra.fr (V.M.); sebastien.elis@inra.fr (S.E.); 2 UMR ISP, INRA 1282, Université de Tours, 37380 Nouzilly, France; ana-paula.teixeira@inra.fr; 3 UMR BDR, ENVA, INRA, Université Paris-Saclay, 78350 Jouy-en-Josas, France; veronique.duranthon@inra.fr; 4 PAIB (Pôle d’Analyse et d’Imagerie des Biomolécules), Plate-forme CIRE (Chirurgie et Imagerie pour la Recherche et l’Enseignement, INRA, Université de Tours, CHRU de Tours, 37380 Nouzilly, France; 5 Profilomic SA, F-92100 Boulogne-Billancourt, France; alexandre.seyer@medday-pharma.com

**Keywords:** ovarian follicle, lipids, mass spectrometry imaging, MALDI MS profiling, gene expression, oocyte, granulosa, theca, follicular fluid, bovine

## Abstract

Ovarian follicle provides a favorable environment for enclosed oocytes, which acquire their competence in supporting embryo development in tight communications with somatic follicular cells and follicular fluid (FF). Although steroidogenesis in theca (TH) and granulosa cells (GC) is largely studied, and the molecular mechanisms of fatty acid (FA) metabolism in cumulus cells (CC) and oocytes are emerging, little data is available regarding lipid metabolism regulation within ovarian follicles. In this study, we investigated lipid composition and the transcriptional regulation of FA metabolism in 3–8 mm ovarian follicles in bovine. Using liquid chromatography and mass spectrometry (MS), 438 and 439 lipids were identified in FF and follicular cells, respectively. From the MALDI-TOF MS lipid fingerprints of FF, TH, GC, CC, and oocytes, and the MS imaging of ovarian sections, we identified 197 peaks and determined more abundant lipids in each compartment. Transcriptomics revealed lipid metabolism-related genes, which were expressed constitutively or more specifically in TH, GC, CC, or oocytes. Coupled with differential lipid composition, these data suggest that the ovarian follicle contains the metabolic machinery that is potentially capable of metabolizing FA from nutrient uptake, degrading and producing lipoproteins, performing de novo lipogenesis, and accumulating lipid reserves, thus assuring oocyte energy supply, membrane synthesis, and lipid-mediated signaling to maintain follicular homeostasis.

## 1. Introduction

In dairy cows, lipid metabolism is crucial to maintain proper reproductive function, which is affected by negative energy balance (NEB) occurring during the early postpartum period, due to the elevated mobilization of fat reserves to liberate the energy that is required for milk production [1,2]. Oocyte competence or capacity to support embryo development after fertilization is one of the key factors affected by misbalanced fatty acid (FA) metabolism during the NEB period [3]. Indeed, high concentrations of free FAs in plasma and follicular fluid (FF) during NEB lead to lipotoxicity within the ovarian follicle and impair oocyte quality, thus reducing cow fertility [4]. The impaired lipid composition of FF, which is particularly rich in saturated free FA (C18:0 stearic and C16:0 palmitic acids) in lactating cows, may explain their lower fertility as compared to heifers [5], as well as the seasonal variations of oocyte developmental competence in dairy cows [6].

Oocytes develop and acquire their developmental competence in the ovarian follicle through tight bidirectional communication with follicular somatic cells, with each compartment secreting specific regulation factors [7,8,9,10]. Ovarian morphological and functional organization supports follicular development and oogenesis, protecting the growing oocyte. Inside the pre-antral follicle, epithelial-like granulosa cells (GC) and mesenchymal-like theca cells (TH) face each other across a basement membrane; these mural cells enclose the oocyte, which grows through folliculogenesis. TH cell layers are transpierced by blood microvessels, and since antrum appearance, the follicle is filled with a fluid that is rich in proteins, steroids, and lipids, coming from the blood and secretory activity of follicular somatic cells—TH, GC, and cumulus cells (CC), too [11,12]. CC are the differentiated GC, which are tightly connected and metabolically coupled with an oocyte via gap junctions [13] and form the oocyte cumulus complex (OCC). The interaction between TH and GC is essential for the maintenance of the original architecture and functions of ovarian cells, especially for the synthesis of steroids, which regulates reproductive function, in response to the follicle stimulation hormone (FSH) and luteinizing hormone (LH). The closest follicular environment of the oocyte, including FF, GC, and especially CC, has a crucial impact on the acquisition of oocyte developmental competence [12,14,15,16] and possesses molecular factors that are predictive of oocyte developmental potential [17,18,19].

Energy metabolism significantly varies between the different types of ovarian follicular cells, especially in oocytes and CC, notably relying on different energy sources, including amino acids, pyruvates over glucose, and fatty acids [20,21]. To produce energy, oocytes use lipid reserves, besides the glucose derivate supplied by CC [22]. 

Free FAs are the major blocks for the synthesis of glycerolipids, such as triacylglycerids (TG), which are mainly used for intracellular energy storage, forming together with cholesterol esters (CE) intracellular lipid droplets (LDs) as reservoirs of caloric reserves. LDs are also caches of FAs and sterol components that are needed for the biogenesis of the membranes [23]. The cholesterol that is used by ovarian cells is derived from either the cellular uptake of plasma lipoproteins, which contain cholesterol, or de novo synthesis [24]. Free FAs and cholesterol form the structural basis for the synthesis of paracrine hormones such as prostaglandins and steroids, which play crucial roles in female reproduction, with particular involvement in the ovulation process [25] and the pre-ovulatory maturation of OCC in bovine [26]. Three lipid families are crucial components of the biological membrane structure: glycerophospholipids containing diacylglycerols (DG) in their hydrophobic part; sphingolipids (SL) with a ceramide as the hydrophobic part, and sterols (ST)—the major non-polar lipids [27]. Hydrolysis of the glycerolipids and SL produces messenger lipids such as DGs, ceramides, unsaturated FAs, and lysophosphatidic acid (LPA), and other membrane lipids may have signaling functions [28].

In the ovary, circulating free FAs are transported from blood as complexes with albumin inside the ovarian follicle, and should be metabolized to form de novo membranes or used as energy sources for the oocyte [21,29]. It is known that in somatic cells, captured FAs are esterified into TG and cholesterol esters (CE), and thus could be stored as neutral lipids in lipid droplets (LDs) for further use [23]. Ovarian follicular somatic cells, in particular TH and GC, receive and metabolize nutritive molecules, including lipids in lipoprotein form from FF [20], and transform them either for storage in LDs, or the building of a membrane bilayer, ATP production, or signaling molecules.

Although lipids are an integral part of all of the biological membranes and universal energy reserves, the distribution of lipids in mammalian ovaries is not homogenous, as was detected by mass spectrometry imaging (MSI). In porcine ovary, the MSI of lipids was performed using matrix-assisted laser desorption/ionization time-of-flight mass spectrometry (MALDI-TOF MS), which clearly discriminated stroma and ovarian follicles through ovarian sections with a particularly specific distribution of glycerophospholipids, sphingolipids, and cholesterol derivatives between somatic cells (GC/TH) and FF inside the follicles [30]. In mice ovary, the localization and intensity of several lipids, including phosphatidylinositol (PI 38:4) and arachidonic acid (C20:4), varied between preovulated and post-ovulated stages [31].

MALDI-TOF MS profiling is a technique that allows the direct detection of the endogenous molecules in a relatively wide mass range beginning from 100 Da to 25,000 Da (ions of lipids, or peptides and proteins), in scarce biological samples. In bovine, this technique was used to analyze lipid fingerprints in oocytes [32,33,34], CC [35,36], and FF [37].

The enzymatic pathways involved in lipid transformations were studied preferentially in lipogenic organs such as adipose tissue [38], liver [39], and also in cancer cells [40]. The proteins that are involved in FA metabolism may have tissue specific distribution, as shown for FA binding proteins encoded by numerous *FABP* genes, with each one preferentially expressed in different tissue [41]. The importance of triacylglycerol and FA metabolism in the oocyte–cumulus complexes and the crucial role of FA oxidation for oocyte quality in mouse was underlined in the review by Dunning et al. [42]. In bovine, until now, the studies reporting on the expression of lipid metabolism-related genes in ovarian cells were focused on only a few key enzymes in oocytes, CC, and GC [35,43,44,45,46,47]. Expression of the main FA transforming enzymes involved in lipogenesis, lipolysis, FA oxidation, and FA transport has revealed active lipid metabolism in the oocyte and surrounding CC and GC. Besides, both lipid synthesis and FA oxidation are involved in the regulation of GC proliferation and steroidogenesis [46]. FA oxidation in CC was crucial for oocyte maturation in bovine [35,44]. However, the mechanisms through which somatic ovarian follicular cells metabolize lipids for oocyte energy supply to promote oocyte quality, and genes regulating lipid metabolism in ovarian follicles, are still poorly elucidated.

The objective of this study was to investigate the lipid composition and transcriptional regulation of FA metabolism in bovine ovarian follicles. We focused our analysis on follicles between 3–8 mm in diameter, which are routinely used for recovering the oocytes for in vitro embryo production technologies in bovine [48]. Lipid identification and their distribution in ovarian follicular compartments (FF, oocyte, CC, GC, and TH), coupled with the gene expression analysis of lipid metabolism-related genes in follicular cells, may enlighten these mechanisms.

## 2. Results

### 2.1. Analysis of Lipids in Bovine Ovarian Follicular Cells and Fluid 

#### 2.1.1. Analysis of Lipids by Nile Red on Ovarian Sections

**Figure 1:**
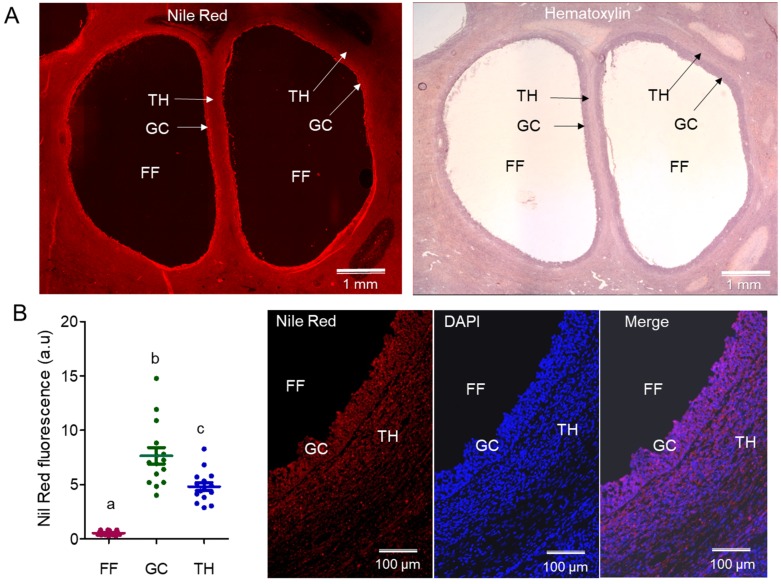
Quantification of lipids in bovine follicular compartments by Nile red fluorescence (NRF). (**A**) Nile red staining (left picture) and hematoxylin coloration of ovarian section (right picture). (**B**) Scatter plot represents Nile red fluorescence quantification in follicular fluid (FF), granulosa cells (GC), and theca cells (TH) in 15 follicles with an average size of 5.5 mm. Different letters mean significant difference at *p* < 0.05, (Kruskal–Wallis test and Dunn’s multiple comparison test). Representative region of the follicle stained with Nile Red (lipids) and DAPI (DNA) is shown.

Nile red fluorescence (NRF) was analyzed on ovarian sections in the equal surface of FF, TH, and GC layers. NRF was more intensive in the cellular compartments of ovarian follicles than in FF (intensity of red fluorescence correlates with a quantity of neutral lipids) (Figure 1A). Normalized NRF intensity was compared between TH, GC, and FF compartments in 15 follicles (5.49 ± 0.28 mm in diameter) from seven ovaries (Figure 1B). Average NRF intensity was significantly lower in FF (0.53 ± 0.05 arbitrary units, au) as compared with GC and TH layers (7.65 au ± 0.75; 4.81 au ± 0.37, respectively). NR staining in the GC layer was significantly higher than in TH layer (*p* < 0.05).

#### 2.1.2. Lipid Composition of Follicular Cells and Fluid

Using liquid chromatography coupled with high-resolution MS (LC/HRMS), 438 different lipids were formerly identified in FF and 439 were formerly identified in the follicular cells of the same ovarian follicles (Supplementary data, Table S1). All of the detected lipids belonged to five families and 21 classes (Table 1).

**Figure 2:**
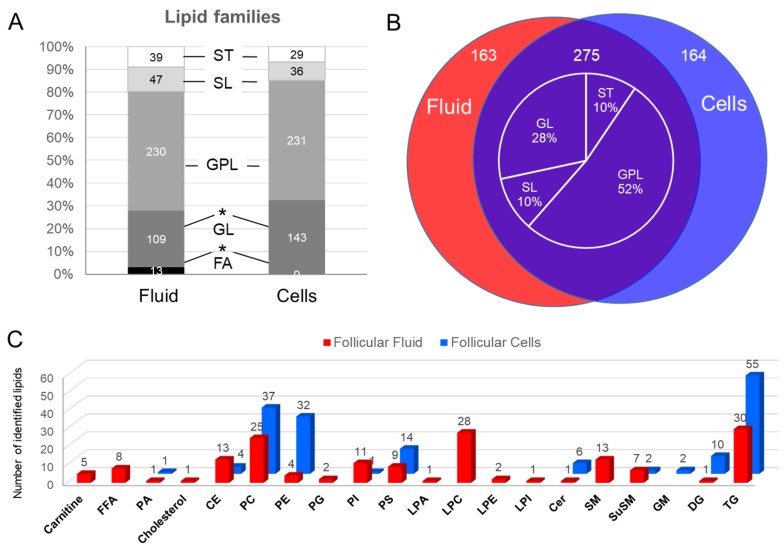
Identification of lipids in bovine 3–8 mm follicles by liquid chromatography coupled with high-resolution MS (LC/HRMS). (**A**) Family distribution of all of the lipids detected in follicular cells and fluid. * indicates significant difference (*p* < 0.05, Chi-squared test). (**B**) Venn diagram presents all of the lipids identified in follicular fluid (FF, red circle) and follicular cells (blue circle). Distribution by families of 275 common lipid species is shown. (**C**) Distribution by classes of the lipids, detected either in follicular cells only (164 species, blue bars) or in FF (163 species, red bars). Number of identified lipids are indicated above the bars. ST—Sterols; SL—Sphingolipids; GPL—Glycerophospholipids; GL—Glycerolipids; FA—Fatty acyls; FFA—Free fatty acid; PA—Phosphatidic acid; CE—Cholesteryl esters; PC—phosphatidylcholines; PE—phosphatidylethanolamines; PG—phosphatidylglycerols; PI—phosphatidylinositols; PS—phosphatidylserine; LPA—Lyso-Phosphatidic acid; LPC—Lyso-phosphatidylcholins; LPE—Lyso-phosphatidylethanolamine; LPI—Lyso-phosphatidylinositols; Cer—Ceramides; SM—Sphingomyelins; SuSM—Sulfoglycosphingolipids; GM—Gangliosides; DG—Diacylglycerols; TG—Triacylglycerols.

Among the five lipid families, detected in FF and follicular cells, similar proportions of sterols (ST, 8.9% and 6.6%, respectively), sphingolipids (SL; 10.7% and 8.2%, respectively) and glycerophospholipids (GPL; 52.5% and 52.6%, respectively) were observed in both compartments (Figure 2A). A significant difference was reported in fatty acyls (FA family), which represented about 3% of the lipids in FF, but was not detected in follicular cells, and in the glycerolipid family (GL), which represented 24.8% of the lipids in FF and 32.6% of the lipids in follicular cells (*p* < 0.05).

From all of the identified lipids, 275 lipid features were common between follicular cells and FF; 164 and 163 features were only detected in either follicular cells or FF, respectively (Figure 2B). Among the common lipids, 52% were GPLs represented mainly by phosphatidylcholine (PC) and phosphatidylethanolamine (PE) (37.1% and 13.8% of total lipids) and two lyso-phosphatidylcholine (LPC). GLs, representing 28% of common lipids, were mainly TG (77 species). These lipids mostly have long carbon chains. Free cholesterol, 25 CE and 23 sphingomyelin (SM), were also identified in both compartments. Lipid species, which were detected either only in FF (163 lipids) or in follicular cells (164 lipids), showed different class distribution (Figure 2C). Five carnitine isoforms, eight free FA, hydroxycholesterol, two phosphatidylglycerols (PG), 32 lyso-glycerophospholipids (28 lyso-phosphatidylcholine (LPC), LPA, two phosphatidylethanolamine (LPE) and lyso-phosphoinositol (LPI)) and 13 SM were identified in FF only. In the cells, neither free FA nor was a specific lyso-GPL identified. Also, CE and PI lipid classes were more represented in FF, whereas more DG, TG, PE, PC, and PS were detected in follicular cells compared to FF. 

#### 2.1.3. Mass Spectrometry Imaging of Lipids in Ovarian Follicle

**Figure 3:**
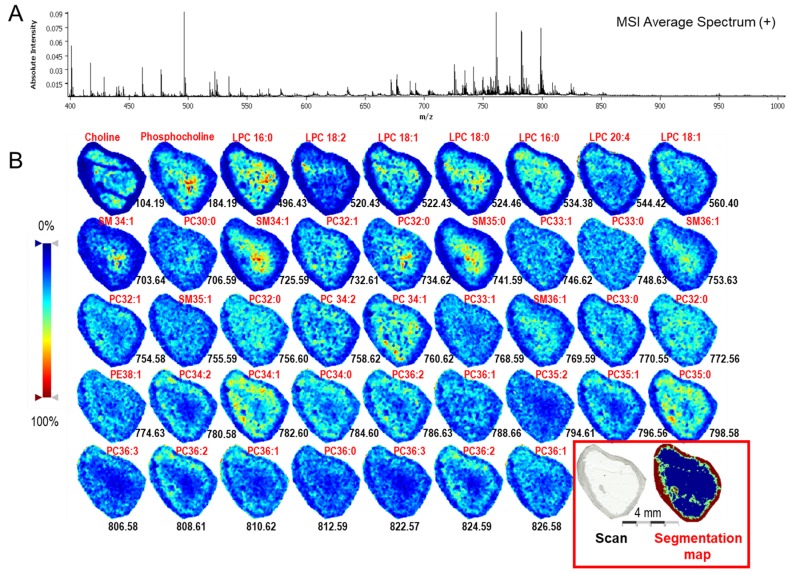
High-resolution mass spectrometry imaging (MSI) of individual bovine follicles. (**A**) Average spectrum of molecular species in the 100–1000 *m*/*z* range. (**B**) Ion density maps of detected lipids in individual follicles. Spatial segmentation map was obtained by hierarchical clustering using the bisecting *k*-means algorithm. Lipid identification was performed by LC–HRMS and/or by direct infusion and MS/MS. LPC—Lyso-phosphatidylcholine; PC—Phosphatidylcholine; SM—Sphingomyelin.

MALDI-TOF mass spectrometry imaging (MSI) performed at 25-µm resolution on a bovine ovarian section allowed the direct mapping of lipids inside the antral follicles. In total, an average spectrum showed 248 molecular species (*m*/*z* 104–827, supplementary data, Table S2) with different intensities throughout the follicle surface (Figure 3A). Both protonated species ([M + H]^+^) and salt adducts species (sodium [M + Na]^+^ and potassium [M + K]^+^) were detected. The identification of lipids by mass spectrometry from liquid extraction surface analysis LESA and classical lipid extracts from FF and cells allowed the characterization of 44 features, which belong mostly to PC, SM, and LPC classes (Table S2). Using MSI, the distribution of identified lipids, which were presented as density maps, is shown in Figure 3B; details of their identification are shown in Table A1 (Appendix A). Several lipid ions were mostly located on the internal part of the follicles, representing FF, as different LPC (*m*/*z* 496.43 LPC 16:0; *m*/*z* 522.43 LPC 18:1; *m*/*z* 524.46 LPC 18:0), SM (*m*/*z* 725.59-SM (d18:1/C16:0); *m*/*z* 741.59-SM (d18:0/C17:0), or PC (*m*/*z* 760.62-PC 34:1). Others lipids were more abundant in the external part, representing follicular cell layers –TH and GC. This is the case for several PC (*m*/*z* 780.58 PC 35:1; *m*/*z* 796.56-PC 35:2, 808.61-PC 39:5). Many lipids were located on both the fluid and cellular parts of the follicle, for example PC 34:0 (*m*/*z* 748.63) and PC 32:0 (*m*/*z* 772.56).

From the MSI sequences, unsupervised hierarchical clustering was performed, in which similar spectra were grouped using multivariate statistical analysis. The created segmentation map using the major clusters was taken for molecular reconstructions of the ovarian follicle for comparison with a histological image. An MSI segmentation map (Figure 3B) demonstrated that the lipid profiles were different between FF and the wall of the follicle; moreover, the mural cellular part of the follicle showed the segmentation into the two layers.

#### 2.1.4. MALDI-TOF Profiling and Lipid Identifications in Follicular Compartments

**Figure 4:**
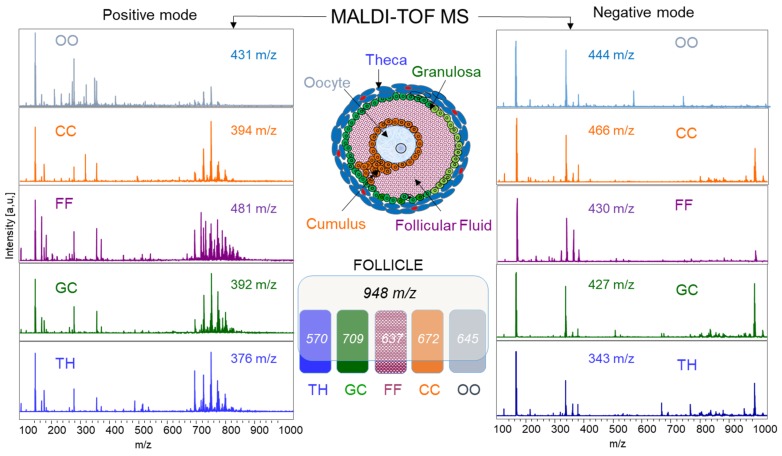
MALDI-TOF profiling performed on isolated follicular compartments. Examples of spectra and number of peaks obtained from individual oocyte (OO), cumulus cells (CC), follicular fluid (FF), granulosa cells (GC) and theca cells (TH) are shown for both positive (left) and negative (right) ion modes. After alignment of spectra from all of the compartments, 948 *m*/*z* were detected from the average spectrum. The number of common peaks between the compartments and the whole follicle are indicated in white italics.

Lipid profiles generated by MALDI-TOF MS on the cellular and fluid compartments of individual follicles were compared between the oocyte, CC, GC, TH, and FF samples (*n* = 12 per condition). The size range of the analyzed individual follicles was 4.2–7.6 mm in diameter. The total lipid profile from each sample included the spectra acquired in both the positive and negative ion modes. Examples of such spectra and a number of peaks detected in the 100–1000 *m*/*z* range in the positive and negative ion modes for each compartment are shown in Figure 4. In the positive mode, more peaks in the 600–800 *m*/*z* range were detected, compared to the negative ion mode, where more peaks in the *m*/*z* 300–400 range were detected in all of the compartments. Visibly, spectra from OO, CC, GC, TH, and FF looked different one from another, in both peak number and relative intensity.

In order to compare the lipid profiles between the follicular compartments, all of the spectra were aligned, and the peaks were detected from the average spectra in both ion modes, bearing in total 948 peaks (462 *m*/*z* and 486 *m*/*z* in the positive and negative ion mode, respectively). 

In order to identify the peaks observed by MALDI-TOF MS profiling, different lipid extracts from the follicular cells and fluid were analyzed by high-resolution mass spectrometry using two strategies as LC-MS and direct infusion for MS/MS structural analyses. All of the identified lipids are presented in Supplementary Tables S3 and S4. For the positive and negative modes, 120 and 62 *m*/*z* were identified, respectively. In the positive mode, most of the identified ions were PC (52.3%), LPC (20.3%), and SM (18.8%). FAC, DG, and TG represented 2.3%, 1.6%, and 4.7% of the identified peaks, respectively. From the negative ions, 54.8% of the identified *m*/*z* were annotated PE, whereas the PI and PS classes each represented 14.5% of the molecular species. FFA and sulfoglycosphingolipid (SuSM) represented 9.7% and 4.8% of identified peaks, respectively.

Normalized peak height values (NPH) of each molecular species were measured for all of the samples. The coefficients of variation of MALDI MS profiling were calculated from all of the peaks and ranged from 15.8% to 32.9% in different tissues (Table A2, Appendix A); mean CV% values were 22.6% and 20.4% for the positive and negative ion modes, respectively.

**Figure 5:**
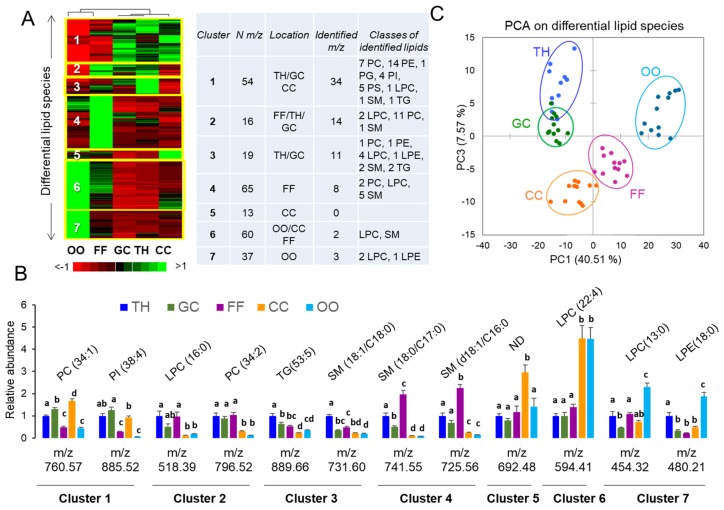
Differential analysis of lipids in bovine TH (theca), GC (granulosa cells), FF (follicular fluid), CC (cumulus cells), and oocytes (OO) by MALDI-TOF MS lipid profiling. (**A**) Heat map representation of differentially abundant lipids (ANOVA, *p* < 0.05). Enclosed table showed the number of peaks per cluster, their specific localization to the ovarian compartment, and the main lipid classes of the identified differential lipids. (**B**) Examples of identified differentially abundant lipids between ovarian compartments (*n* = 12 per compartment). Different letters indicate significant differences (*p* < 0.05, Tukey test for multiple pairs comparison). (**C**) Discrimination of ovarian follicular compartments by their lipid profiles, by principal component analysis. LPE—Lyso-phosphatidylethanolamine, LPC—Lysophosphatidylcholine, PC—Phosphatidylcholine, PE—Pphosphatidylethanolamine, PI—phosphatidylinositol, SM—Sphingomyelin, TG—Triacylglycerol.

The differential analysis of lipid composition was performed by comparison of the mean NPH values between TH, GC, FF, CC, and the oocytes (*n* = 12 per each follicular compartment). By ANOVA, 338 and 196 differentially abundant *m*/*z* species were detected in the positive and negative mode, respectively (*p* < 0.05; maximum fold change >three). All of the NPH values and statistics are shown in Supplementary Data Table S5. The NPH log-values (relative abundance) of 264 of the most abundant differential peaks was presented here as a heat map (Figure 5A).

Lipid features were distributed in seven clusters according to their relative abundances; each cluster represented specific follicular compartment abundance of lipid species. Globally, clusters 1, 2, and 3 (54 *m*/*z*, 16 *m*/*z* and 19 *m*/*z*, respectively) contained the lipids, which were more abundant in somatic follicular cells. Cluster 4 included the lipids that were more abundant in FF. Peaks from clusters 6 and 7 (60 *m*/*z* and 37 *m*/*z*, respectively) were mostly represented in the oocyte. Thirteen features from cluster 5 were preferentially detected in CC. Lipid identification allowed the annotation of 66.3% of differential lipid species from clusters 1–3, which were mainly represented in somatic cells, and 7.2% of the lipids from clusters 4–7, representing FF and OCC (Supplementary Data, Table S6). Among the 120 lipids that were more abundant in the OCC, only several features were identified: three LPC (LPC 13:0, LPC 14:1, and LPC 22:4), SM 32:1, and LPE 18:0. The lipids that were abundant in FF (clusters 2 and 4) belonged to the PC, LPC, and SM lipid classes. TH-specific cluster 3 included the LPC, PC, SM, PE, CE, and TG classes of lipids. The GC lipid profiles were more similar to other somatic cells, and the mostly abundant lipids in follicular somatic cells (clusters 1 and 2) were mainly PC, but also PE, PS, PI, and several lipids belonging to the SM and TG classes. Several peaks may have two or more identifications (Supplementary Data, Table S6); here, we presented the identification with the lowest delta to the exact *m*/*z* value. Examples of differentially abundant identified lipids in each cluster are shown (Figure 5B).

Principal component analysis was performed using NPH values of the most differential lipids, and it clearly discriminated TH, GC, CC, oocyte, and FF compartments (Figure 5C).

### 2.2. Analysis of Gene Expression in Bovine Ovarian Follicular Cells 

Among more than 22,000 genes analyzed using bovine microarray, 11,761 genes showed significant difference of expression between follicular cells (TH, GC, CC) and the oocyte (*p* < 0.05, Benjamini*–*Hochberg (BH) correction). Gene set enrichment analysis (Supplementary Table S7) revealed that differentially expressed genes (DEGs) were significantly enriched in gene ontology (GO) terms as metabolic pathways (*p* = 0.0004) and in GO terms related to the ribosome, cell cycle, peroxisome, spliceosome, and focal adhesion (*p* < 0.001). Among numerous other terms, FA metabolism, FA degradation, proteasome, progesterone-mediated oocyte maturation, pentose phosphate pathway, and pyruvate metabolism were also significantly enriched in the list of DEGs (*p* < 0.05). DEGs were involved in several significantly enriched pathways, such as the mTOR, p53, TGF-beta, PI3K-AKT, FoxO, ErbB, and MAPK signaling pathways (*p* < 0.05).

**Figure 6:**
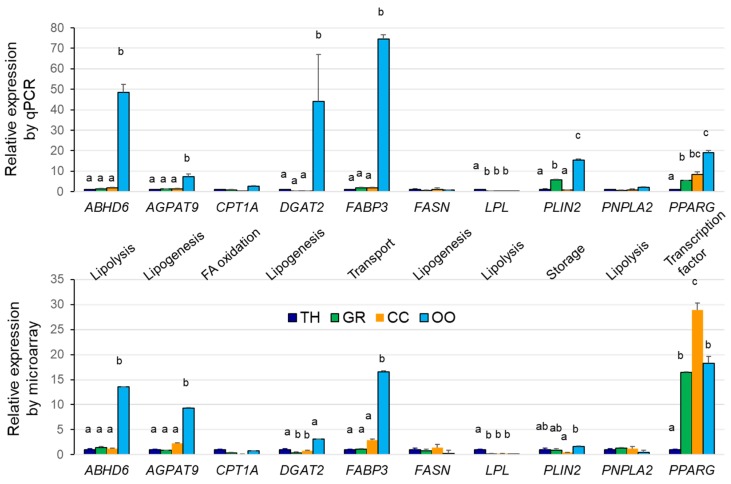
RT-qPCR validation of microarray gene expression analysis. Graphs are presented as the mean+/SEM of normalized expression values of several independent RNA samples per tissue (*n* = 4 for microarray, and *n* = 6 for RT-qPCR analysis). Different letters indicate difference at *p* < 0.05 (ANOVA, Tukey post-hoc test). *ABHD6*—Abhydrolase Domain Containing 6, *AGPAT9*—1-Acyl-Sn-Glycerol-3-Phosphate O-Acyltransferase 9, *CPT1A*—Carnitine Palmitoyltransferase 1A, *DGAT2*—Diacylglycerol O-Acyltransferase 2, *FABP3*—Fatty Acid Binding Protein 3, *FASN*—Fatty Acid Synthase, *LPL*—Lipoprotein Lipase, *PLIN2*—Perilipin 2, *PNPLA2—*Patatin Like Phospholipase Domain Containing 2, *PPARG—*Peroxisome Proliferator Activated Receptor Gamma.

The results on gene expression obtained by microarray hybridization were validated by real-time PCR analysis on several genes that were involved in lipid and FA metabolism. Overall, the same expression patterns were observed using these two approaches for most of the analyzed genes (Figure 6). As shown, *FASN* (fatty acid synthase), *CPTA1* (involved in FA oxidation), and lipase *PNPLA2* are expressed similarly in all of the compartments. In contrast, the other genes that are involved in GL synthesis (*AGPAT9* and *DGAT2*), lipid storage (*PLIN2*), and FA transporter FABP3 were more expressed in oocytes. Transcriptional factor *PPARG* was strongly expressed in oocytes and more abundant in GC and CC compared to TH. 

**Figure 7:**
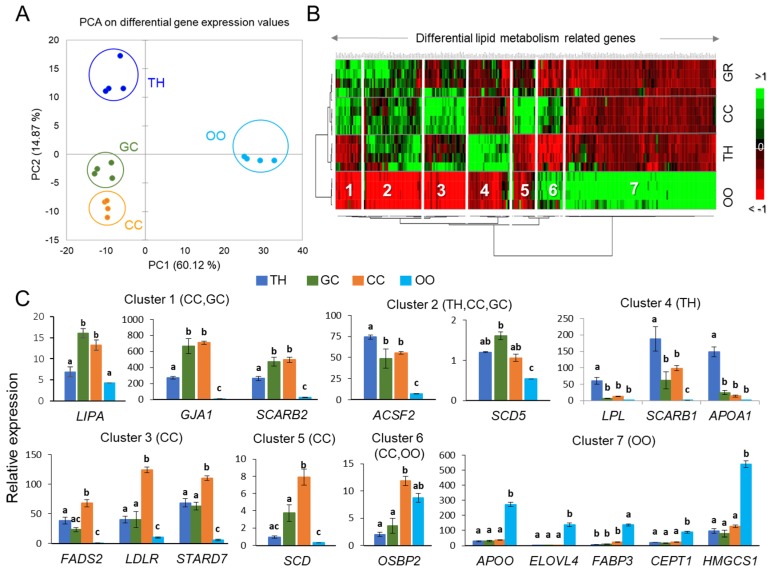
Differential analysis of gene expression in bovine theca (TH), granulosa cells (GC), cumulus cells (CC), and oocytes (OO), using microarray hybridization (*n* = 4 per each cell type). (**A**) Discrimination of ovarian follicular compartments by expression profiles of lipid metabolism-related genes, using principal component analysis. (**B**). Heat map representation of differentially expressed genes (*p* < 0.05, Benjamini–Hochberg correction) related to lipid metabolism. (**C**) Examples of differentially expressed genes between ovarian follicular compartments. Bars are the mean values of four independent replicates +/− SEM. Different letters mean significant difference at *p* < 0.05 (ANOVA, Tukey test for multiple comparisons). *LIPA*—Lipase A; *GJA1—*Gap Junction Protein Alpha 1; *SCARB2—*Scavenger Receptor Class B Member 2, *ACSF2*—Acyl-CoA Synthase Family Member 2, *SCD5*—Stearoyl-CoA Desaturase 5, *SCD*—Stearoyl-CoA Desaturase, *OSBP2*—Oxysterol Binding Protein 2, *LPL*—Lipoprotein Lipase, *SCARB1*—Scavenger Receptor Class B Member 1, *APOA1*—Apolipoprotein A1, *FADS*2—Fatty Acid Desaturase 2, *LDLR*—Low Density Lipoprotein Receptor, *STARD7*—StAR (Steroidogenic Acute Regulatory Protein) Related Lipid Transfer Domain Containing 7, *APOO*—Apolipoprotein O, *ELOVL4*—Elongation Of Very Long Chain Fatty Acids Protein 4, *FABP3*—Fatty Acid Binding Protein 3, *CEPT1*—Choline/Ethanolamine Phosphotransferase 1, *HMGCS1—*3-Hydroxy-3-Methylglutaryl-CoA Synthase 1.

In order to decipher the involvement of different follicular cells in the regulation of lipid metabolism inside the follicle, we analyzed the mRNA expression of the genes, which controls the main steps of the lipid and steroid turnovers. The normalized expression values of 468 genes that were selected by their involvement in energy metabolism, steroidogenesis, were compared between TH, GC, CC, and the oocytes from follicles of 3–8 mm. Among them, 432 genes were found to be differentially expressed. Gene description, expression values, and statistics are reported in the Supplementary Data, Table S8. Principal component analysis (PCA) was performed using the expression values of these genes, and clearly discriminated follicular cells (Figure 7A). Hierarchical clustering regrouped the most discriminant DEGs; a heat map representation of this analysis is shown in Figure 7B. From the heat map, seven clusters were defined according to gene expression patterns; Table 2 presents cluster composition. The representative genes for each cluster are shown in Figure 7C.

Clusters 1 and 2 represent the genes that were overexpressed in somatic cells (GC/CC and TH/GC/CC, respectively). Among them, there were a number of genes regulating steroidogenesis and the synthesis of cholesterol (cytochromes P450 and others), genes involved in the synthesis of Acyl-CoA (*ACFS2*) and different membrane lipids and TG (*SCD5*), genes involved in lipid degradation as cholesterol esterases *(LIPA*, *LCAT)*, phospholipases A and C *(PLA2G1B*, *PLCG1)*, and di- and monoacylglycerol lipases (as *DAGLB*, *ABDH3*, etc.). In addition, these two clusters enclosed the genes involved in FA oxidation (*ACAD10*, *ACSS3*) and lipid transporters *(CD36*, *OSBPL7*, *CSP2*, *SCARB2)*. Genes coding for apolipoproteins (*APOA2*, *APOC3*, *APOL3*, *APOM*), gap-junction proteins (*GJA1*) and *GPX* peroxidases showed the same expression pattern. These clusters were significantly enriched in the following biological process GO terms: phospholipid scrambling, cholesterol binding, ketone body, and bile-acid biosynthetic processes, acyl-CoA, glycerophospholipid biosynthetic processes, sterol transport, FA oxidation, unsaturated FA metabolism, and cellular response to oxidative stress. Enriched pathways included arachidonic acid metabolism, PPAR signaling, and glycerophospholipid metabolism. Genes with more abundant expression in CC constituted clusters 3 and 5. Globally, the genes of these clusters are also involved in cholesterol and steroid transport and metabolism (*LDLR*, *STARD7)*, regulator of lipid synthesis (as *FADS2*, *SCD*), lipolysis (as *ABHD16A*), and in FA oxidation (*ACADVL*). Enriched GO terms included FA beta-oxidation using acyl-CoA dehydrogenase, steroid, lipid, glycerophospholipid, and monocarboxylic acid biosynthetic processes; FA oxidation, FA catabolic process, the regulation of cholesterol metabolic process. Enriched pathways were related to glycerophospholipid and FA metabolism, PPAR, phosphatidylinositol and phospholipase D signaling, choline metabolism, and FA degradation. 

The genes that are overexpressed in TH cells (cluster 4) are particularly involved in cholesterol import *(SCARB1*, *STARD5*), bile acid metabolism (*CH25H*), HDL-mediated lipid transport (*APOA1*, *PLTP*, *SCARB1*), lipid degradation (*LPL*, *PLIN5*), and steroidogenesis (*CYP27A*, *CYP7B1*, *TM7SF2*). GO terms enriched in the TH-specific cluster were related to sterol import, the regulation of sequestering of triglycerides and lipid storage, monocarboxylic acid and bile acid biosynthetic processes, and sterol metabolic process. Enriched pathways were related to PPAR signaling, glycerolipid and primary bile metabolism, phosphatidylinositol, and phospholipase D signaling systems.

Cluster 6 represents the genes that are particularly abundant in oocyte-CC complexes such as intracellular lipid transporter *OSBP2*, and cluster 7 enclosed the genes that are overexpressed in the oocytes. Among the genes of this cluster, we found numerous enzymes transforming lipids such as FA elongases (*ELOVL* genes), desaturases (such as *FADS3*), different phospholipases (phospholipases A and D) and their regulating proteins (*PLAA).* Also, these clusters include the genes that are involved in glycerophospholipid and triacylglycerol synthesis (for example *SPTLC1*, *DGAT2*, *AGPAT*, *GPAT*…), cholesterol biosynthesis (*HMGCS1*), and sphingolipid synthesis (*SPTLC2*, *ACER*, *CERS2*…). The oocytes overexpress gene coding for lipid transporters, as FA and oxysterol-binding proteins (*FABP* and *OSBPL* genes, respectively), and apolipoprotein *APOO*. In addition, these clusters enclosed numerous genes that are related to FA mitochondrial and peroxisome oxidation and genes involved in cholesterol and steroid metabolism, as well as in oxidative stress response (*SOD* genes). The most enriched biological process GO terms in clusters 6 and 7 include glycerophospholipid and phosphatidylinositol biosynthetic processes, long-chain fatty-acyl-CoA and acyl-CoA metabolic processes, FA elongation (saturated and unsaturated), FA and lipid phosphorylation, sphingolipid metabolic processes, and membrane lipid biosynthetic processes. Among the pathways enriched in oocyte-related clusters, there were phosphatidylinositol and inositol lipid-mediated signaling, PPAR signaling, FA elongation, FA degradation; GL and GPL metabolism; peroxisome, choline and sphingolipid metabolism; and insulin receptor signaling pathway. 

## 3. Discussion

In the present work, for the first time, using liquid chromatography coupled to high-resolution mass spectrometry, we identified several hundred lipids representing 21 lipid classes in cellular and fluid compartments of bovine ovarian follicles running the terminal part of the folliculogenesis. In addition, MALDI-MS profiling was, for the first time, used to compare lipid fingerprints in the different cell types of individual ovarian follicles. Then, 197 molecular species in the range of 250–1000 *m*/*z* were formerly identified in bovine follicular cells, and these identifications implemented the list of lipids that were already annotated in bovine oocytes and cumulus cells [32,33,36,49,50]. By these two approaches, we could identify the major lipids, which were differentially abundant among the somatic compartments: TH, GC, CC, and the oocyte. Although only 1/5 part of *m*/*z* peaks detected in MALDI-MS spectra was precisely identified, these analyses coupled to MS imaging enabled localizing the most abundant lipids to ovarian follicular structures.

The majority of identified follicular lipids are known to be involved either in membrane synthesis or energy storage (CE, SL, GPL, GL). In fact, we have analyzed tertiary follicles where somatic follicular cells yet proliferate, and the oocytes, which were close to fully grown and potentially ready to resume meiosis, continued to acquire developmental potential by accumulating molecular reserves [51]. At that stage, the follicles may have strong requirements in energy and building blocks for membranes biosynthesis. During follicular growth up to ovulation, constant bi-directional interplay occurs between the oocyte and surrounding follicular cells. Indeed, the oocyte secretes specific factors GDF9 and BMP15, and thus stimulates follicular cells to proliferate and secrete steroids and other factors, which, in turn, regulate oocyte growth [52,53] and protect it from the harmful environment [54]. Follicular fluid contains molecular factors that were both exuded from blood and follicular secretion [55], and therefore acting as a buffer against adverse blood influence and as a medium of molecular exchanges between follicular cells. Significant proportions of the follicular fluid lipids that were identified here (FA, Cer, DG, lysophospholipids, LPI, LPE, LPC, ..) might exhibit mediator functions [28,56]. FF extracellular vesicles such as exosomes [57] have very specific lipid composition [58], and may participate in these communications inside the follicle.

In order to analyze the lipid distribution through the ovarian follicle, molecular imaging and phenotyping technologies were performed. MSI on bovine ovarian sections allowed the ionization of numerous lipids, including PC, LPC, SM, and choline molecules. MSI segmentation map clearly discriminated FF from TH and GC layers, similarly to the MSI of porcine ovarian sections [30]. This could be explained by different lipid compositions; for example SM, LPC, and choline molecules were preferentially detected in FF, but also by the different ionization capacity of lipids from either liquid or cellular compartments. The identified PC features were mainly localized to both follicular walls and fluid by MSI. Among the common identified lipids, PC represented 37%, and among the identified MALDI-MS *m*/*z* peaks, PC represented more than 50%. PC are the major components of cellular membrane lipids with well-established roles in differentiation and cell proliferation [27]. Therefore, PC localized to the follicular wall may be associated to membrane structures in TH and GC.

MALDI-MS lipid profiling confirmed the higher abundance of PC, as well as other membrane lipids such as PE and PS that feature in GC and TH cells as compared to FF. Overall, the FF lipid profile significantly differed from that of the follicular cells, as was expected from the non-cellular compartment. The TH and GC profiles were partially overlaid, as shown by the principal component analysis of MALDI fingerprints. In fact, although TH and GC are separated by the basal membrane, they are metabolically coupled; thus, many lipids were shared between these layers. However, the increased abundance in several LPC, LPE, SM, and TG in TH compared to GC were sufficient to discriminate these two cell types. A similar approach was performed on human lung where MSI confirmed the specific distribution of three phospholipids (PE, PI, and PG) in different regions of lung slice, illustrating the lipidome differences between alveolar macrophages, bronchial epithelial, and alveolar type II pulmonary cells, which were previously shown by LC-MS/MS [59]. The specific lipid profiling of TH probably reflects its functional structure, which is rich in steroid-secreted cells, blood capillaries, and collagen [60], compared to GC, which is more homogeneous and has smoother tissue.

MSI was not sufficiently resolute to discriminate GC and CC; however, MALDI-MS lipid fingerprints showed significant differences between these cells. Although CC was derived from the GC, the specific functions of CC are conditioned by their physical and metabolic coupling with the enclosed oocyte [61]. Here, GC shared the most of the lipids with other compartments; however, in CC, several not-identified features were particularly abundant and formed a CC-specific cluster. In our previous study, a comparison of GC and CC lipid fingerprints revealed that two LPC, 12 PC, and 12 SM features were up-regulated in CC whereas several features including LPC, four PC, two PE, PS, and Cer were more abundant in GC [62]. The differences between GC and CC lipid concentrations likely reflect the functional differences between these cells [63]. 

Lipid concentrations in follicular compartments may be predictive for oocyte competence. Indeed, the concentration of several TG, PG, PI, PE and lysophospholipids LPE, LPI, and LPC in human FF demonstrated a strong potential association between the differentially elevated lipids and increased success rate of in vitro fertilization/intracytoplasmic sperm injection outcomes [64]. While CC are physically and metabolically coupled with enclosed oocytes, the MALDI-TOF MS lipid profiling of these cells may also reflect oocyte competence. The differential abundance of several PC was observed in the bovine CC enclosing the oocytes with higher developmental potential after FA (22:6 n-3) supplementation [36]. In human, the MALDI-TOF lipid profiles of CC showed a different abundance of PC species in the pregnant group and PE, PS, and PI features in the non-pregnant group after ICSI [65]. Lipid composition also differed between the oocytes with different competence in bovine [33,36,43], but it is difficult to thoroughly evaluate the roles of each feature in this difference. 

Oocytes demonstrated very specific lipid profiles that were distinct from the other follicular compartments; nevertheless, the clustering of differential lipids revealed common features with CC or FF. Among the few identified lipids from oocyte-representative clusters, LPC(13:0) and LPC(18:0) were the most elevated in oocytes, LPC 22:4 was similarly abundant in oocytes and CC, and SM (d32:5) was detected at similar levels in oocytes and FF. Previously, we showed significant variations of several PC, SM, and free FA concentrations in the oocytes during meiotic maturation [34]. This was in line with a decreasing of lipid reserves in mature oocytes as compared to the immature stage [43], and highlighted the profound changes in both membrane composition and intracellular lipid storage during oocyte maturation.

A relative quantity of total lipids in cellular and fluid compartments was measured using Nile red fluorescent dye in situ targeting mainly the neutral lipids, TG, and cholesteryl esters, which are the main components of lipid droplets (LDs) [23]. GC and TH layers demonstrated intensive fluorescence due to the accumulation of LDs, which may serve as an energy reserve and as precursors of the steroid hormones or secondary messengers produced by GC and TH.

Steroidogenesis in ovarian follicular cells is mediated by a number of well-known enzymes such as, for example, steroidogenic acute regulatory protein STAR, cytochrome P450 side chain cleavage CYP450scc, 3-beta-hydroxysteroid dehydrogenase HSD3β, and CYP450 aromatase in theca and granulosa cells [66]. Here, transcriptome analysis confirmed the expression of genes involved in steroid biosynthesis in TH, GC, and CC, as expected. From the same analysis, expression of numerous genes involved in acyl-CoA, FA, and lipid biosynthesis, including the TG, SL, and GPL families, was evidenced in follicular cells, hypothesizing the existence of ovarian de novo lipogenesis in bovine ovary. It was previously reported that CC could produce cholesterol, and this process is regulated by the oocyte secretion of BMP15/GDF9 in mice [67]. The expression of the key enzymes that are involved in FA synthesis was previously reported in porcine TH and GC [30]. In bovine GC, CC, and oocytes, the expression of lipogenic enzymes FASN and ACC was evidenced at the protein level [35,43,46]. Sterol regulatory element binding transcription factor 1 (*SREBF1*) regulates the genes that were involved in cholesterol and FA biosynthesis [68]. Here, it was detected as ubiquitously expressed in follicular cells, and thus may participate in the regulation of lipid metabolism-related genes.

The accumulation of lipids in LDs by follicular cells is regulated by a number of genes that are strongly expressed in GC, TH, and CC, and are involved in triglyceride biosynthesis (for example, *GPAM*, *GPD1*, *SCD5*, and *ACSBG1)*, lipid storage (*SCARB1*, *SCARB2*, *LPL*, *PLIN2*, and *PLIN5*), and glycerophospholipid metabolism (*PLA2G16*, *GPAM*, *ABHD3*, *PLA2G1B*, and *GPD1*). Correspondent proteins participate in lipoprotein degradation and lipogenesis, leading to the formation of intracellular LDs. In humans, GC accumulate more LDs than CC in vivo [69]. However, CC, which are specialized GC, are also able to rapidly accumulate LDs, especially when exposed to a high concentration of free FA [70,71]. Such a rapid transfer of free FA from FF and their accumulation in the LDs of follicular cells protect the oocyte from lipotoxicity, as was shown in bovine CC [54].

TG, CE, and free FA were also largely represented in FF, although there are no LDs. Indeed, total TG concentrations decreased, and total cholesterol increased in bovine FF along with follicular growth from small to large antral follicles [72]. These lipids may be a part of high density lipoproteins (HDL), which are the smallest lipoproteins that can be transported from blood [24]. Follicular cells may also synthetize lipoproteins, as human GC, which produced very low-density lipoproteins [73]. Among the lipids identified in FF, there were many membrane lipids (PC, PE, PI, PS, and lysophospholipids). These neutral lipids may be a part of FF lipoproteins, similar to human plasma lipoproteins, as analyzed by MALDI-TOF MS [74].

TH and GC cells received nutrients from circulating blood, including different lipoproteins and free FA coupled to albumins. In the present study, the expression of FA transporter *CD36* [75] and scavenger receptors *SCARB1* and *SCARB2* for HDL cholesterol esters uptake [76], which was detected in both TH and GC, supports the exogenous origin of a part of FF neutral lipids. In addition, we showed that the lipoprotein lipase *LPL* gene was highly expressed in TH cells, as well as other lipases in both TH and GC, suggesting that lipoproteins may be rapidly metabolized and liberate TG and CEs, which may be further used for energy storage and steroid synthesis. Follicular fluid also contain the debris of floating atretic GC and extracellular vesicles, which are released from cell membranes [77] and are abundant in bovine FF [57]. Therefore, the PC, as the other membrane phospholipids identified in FF, may originate from both lipoproteins and be membrane-derived, similar to extracellular vesicles, including exosomes [58,78].

On the other hand, several genes coding for apolipoproteins A, E, M, L, and O were strongly expressed in GC, TH, and CC. At the protein level, ApoA1 was shown to be produced and secreted by chicken GC [79]. ApoA1 protein was also evidenced by Western blot in the GC of healthy and polycystic ovary syndrome patients [80]. By immunocytochemistry, the ApoA1 protein was revealed in luteal follicular cells in primates [81]. In our previous work, using top–down proteomics, we have identified ApoA2 and ApoC3 proteins in bovine follicular cells (GC and CC) [82,83]. ApoA1, ApoC3, ApoH, and ApoO were identified using differential detergent fractionation multidimensional protein identification in bovine oocytes and CC [84]. Our present work reported the expression of corresponding genes in follicular cells and oocytes. Therefore, it seems that the bovine follicle is capable of not only degrading, but also synthetizing, the lipoproteins.

In addition, eight free FA, three SM, 28 LPC, and 13 cholesteryl ester features were identified in only FF by LC/HRMS. Around 10% of the total fatty acid concentration that is present in FF is complexed to albumin as free FA, and this pool is the most variable, and depends on the metabolic status of the cow [14,71]. Some of the FF lipids, as LPA or unsaturated FA, can activate peroxisome proliferator-activated receptors PPARs, nuclear transcriptional factors that control the expression of genes that function in lipid and carbohydrate metabolism, vascular development, cell proliferation, and cell differentiation [85]. Our study demonstrated that PPARG is strongly expressed in GC, CC, and oocytes, and may therefore regulate numerous lipid metabolism related genes in all of the compartments, as was here revealed by gene ontology analysis.

Sphingolipids are involved in membrane structure stabilization, cell-to-cell recognition, and cell signaling [86,87]. The preferential location of SM and LPC to FF might be also related to extracellular vesicles, which contain these lipids [58], and to lipoproteins, which contain both SM and lysophospholipids such as LPC [74].

The circulating lysophospholipids are membrane components that are necessary to mediate the synthesis of various phospholipids and embed proteins into cell membranes; however, several lysophospholipids such as LPC and LPI were now recognized as hormone-like signaling molecules acting as the ligands of orphan G protein-coupled receptors, and thus considered as intercellular and intracellular lipid mediators [56]. Therefore, LPC in FF might be involved in intrafollicular signaling and cell-to-cell communication.

LPCs that are derived from the PC are catalyzed either by lecithin-cholesterol acyltransferase LCAT with the formation of a cholesterol ester, or by phospholipases A2. According to our data, the genes *LCAT*, *PLA2G16*, *PLA2G7*, and *PLA2G1B* coding for the correspondent enzymes are expressed in TH, GC, and CC, whereas phospholipase A2-activating protein *PLAA* is overexpressed in oocyte. Therefore, we hypothesize that LPC can be produced by follicular cells, and oocyte may influence this process. Integrated to lipoproteins, the LPC may be then secreted to FF, which therefore should contain the LPC originated from both plasma and follicular cells. Reciprocally, LPC and LPE are transformed to PC or PE by specific acyltransferases, and these enzymes *(LPCAT2* and *MBOAT2*, respectively) were also expressed in follicular cells, with significant overexpression in the oocytes. According to MALDI MS profiling, LPC (16:0) and LPC (18:0) were more detected in the oocyte than in somatic follicular cells, and LPC (22:4) were preferentially detected in oocytes and CC. These LPCs may be either imported from the follicular cells, or locally produced. In addition, oocyte expresses genes to transform LPC to PC (*CEPT1*) or produce lipoproteins (*APOO1*). Several lipid transporters are expressed in the oocyte–cumulus complex and indicate possible exchanges of different lipids between these two compartments, as was shown for FABP3 protein [88], or between OCC and FF.

Within an OCC, a bovine full-grown oocyte exercises low transcriptional activity or is transcriptionally silent [89]. Transcripts are accumulated during oocyte growth and then stored as long as protein synthesis is required. When polyadenylation occurred, mRNA could be translated. By our transcriptomic analysis, only polyadenylated mRNA levels were compared between the follicular cells by microarray; therefore, here, detected overexpressed mRNA could be translated in the oocyte at that stage. Although in this article we did not reported data on protein expression, fatty acyl synthase FASN, acetyl-CoA carboxylase ACC, FA binding protein FABP3, and lipid-droplet associated perilipin PLIN2 were detected at protein level in bovine immature oocytes recovered from 3–6 mm follicles [35]. Moreover, among 811 proteins identified by proteomics in bovine oocyte, numerous ones are involved in lipid metabolism [84], and the expression of correspondent genes was detected in the present study. 

Full-grown bovine oocytes contain many lipid inclusions as LDs and ooplasm vesicles, which could be easily detected and quantified using specific fluorescent dyes such as NR or BODIPY [35,43,71]. In cows, a number of intracellular LDs increased during oocyte growth in vivo [90,91] and decreased during in vitro maturation [43]. Significant correlation between the number of LDs in the oocytes and FA composition in FF was reported in bovine; however, no correlation was found between the number of LDs in the oocytes and the expression level of lipid biosynthesis genes *SCD*, *FADS2,* and *ELOVL5* in GC or CC [92]. According to our transcriptomic data, a set of genes that are involved in the synthesis of glycerolipids *(DGAT2*, *GK*, *AGPAT9*, *MBOAT2*, *PPAP2C*, *AKR1B1*, *GPAT2*, *DGKI)*, sphingolipids (*SPTLC2*, *ACER*, *CERS2*), phospholipids PC and PE (*CEPT1*), lipid storage (*PLIN2*), and FA transport (*FABP3*, *FABP5*) are overexpressed in the oocytes compared to other follicular cells. Expression pattern corroborates with a presence of numerous LDs in the oocyte at that stage and suggest strong lipogenic activity, such as the FA elongation and synthesis of very long-chain fatty acyl-CoA, which is more pronounced in oocytes than in other follicular cells. Oocyte expression pattern also supports its capability to intensive lipolysis and FA oxidation activities, which corroborates with the presence, at protein level, of hormone-sensitive lipase (LIPE protein) in immature oocytes and an increase of LIPE phosphorylation and CPT1 protein abundance in bovine oocytes during maturation [35,43].

In addition, transcriptome analysis revealed the role of the oocyte in PI3 kinase signaling and the synthesis of phosphatidylinositol phosphates (PIP) at the plasma membranes because of the overexpression of the genes that are involved in phosphatidylinositol metabolic processes *(CDS1*, *PI4K2B*, *PLA2G12A*, *PIK3CB*, *PIK3R1*, *PIK3C2A*, *PIK3CA*, *PITPNM1*, *PLCG2*, *PIP4K2A*, *PIK3C3*, *PIP4K2B*, *PIP5K1B*, *PI4KB*, *PIP4K2C*, *PIP5K1C…).* Indeed, the hydrolysis of PIP_2_ and the production of inositol trisphosphate is involved in the triggering of oocyte activation after fertilization [93]. In immature oocytes, which were here analyzed, mRNA may be stocked or partially translated. During oocyte maturation in bovine, protein de novo synthesis occurs in parallel with massive mRNA degradation [94,95,96]. It is likely that the accumulation of lipid reserves, together with maternal mRNA and protein stocks, may serve to ensure oocyte maturation, fertilization, and zygote activation. The first rapid embryo cleavages require active machinery to produce cell structures from the maternal stock for at least 16 blastomers before relaying to the embryo genome that activates in 8/16-cells stage embryo in bovine [97]. 

In summary, the provided detailed analysis of lipids and related transcriptional control in the bovine ovarian follicle implies the capacity of bovine follicular cells, especially theca and granulosa cells, to uptake lipoproteins and free FA, metabolize them, and transport lipids. From these blocks, the follicular cells may probably either produce new membrane lipids and lipoproteins or liberate energy through FA oxidation, or constitute lipid droplets as a reserve of energy and precursors of steroid hormones and secondary messengers. The lipid composition of follicular fluid and enclosed oocyte–cumulus complex coupled to a particular gene expression pattern in these cells presumes intensive lipid exchanges between these compartments, and the particular involvement of the oocyte in FA elongation, the synthesis of glycerophospholipids, glycerolipids, and lipid droplet formation. Oocytes thus accumulate lipids and also contain specific metabolic machinery that may be used for energy production, membrane synthesis, and signaling in order to assure meiotic maturation, fertilization, and provide necessary building blocks for the first embryo cells.

Although more investigations at the protein level are required, an analysis of the expression patterns of lipid metabolism-related genes in the follicular cells and the scrutiny of lipid composition in follicular compartments may imply that the ovarian follicle contains complex metabolic machinery for FA uptake and metabolism to assure intrafollicular homeostasis and prepare the oocyte for ovulation. 

## 4. Materials and Methods

### 4.1. Ethics

Bovine ovaries were obtained from a local commercial slaughterhouse; no experiments on live animals were performed.

### 4.2. Chemicals

Unless indicated, all of the chemicals were purchased from Sigma-Aldrich (Saint-Quentin Fallavier, France). LC-MS grade water (H_2_O) and methanol (MeOH) were from VWR International (Plainview, NY, USA). Internal standard mix for lipid identification was purchased from Avanti Polar Lipids, Inc. (Alabaster, AL, USA).

### 4.3. Biological Materials

Bovine ovaries were transported from the local slaughterhouse on ice. Follicles of 3–8 mm in length were accurately aspirated in 50-mL Falcon tubes, avoiding blood contamination, on ice. Follicular cells fell down in the bottom of the tube were recovered and centrifuged for 10 min 5000 *g* at 4 °C. The cell pellets were rinsed twice in cold phosphate buffered saline (PBS). Samples of follicular cell pellets and follicular fluid (FF), separated from cell debris, were frozen in liquid nitrogen and used for lipid extraction and identification using liquid chromatography coupled with high-resolution mass spectrometry (LC-HRMS) or tandem high-resolution mass spectrometry (HRMS/MS).

For mass spectrometry imaging (MSI) and liquid extraction surface analysis (LESA) from ovarian sections, ovaries were frozen in nitrogen stream and cut within no longer than four days for lipid analysis.

For lipid profiling by MALDI–TOF MS, individual follicles were dissected in order to separate FF, scraping off granulosa cells (GC), and isolating theca cell layers (TH) from each follicle. Cells were put into cold PBS, centrifuged, and then the pellets were washed twice in Tris-Sucrose buffer (260 mM sucrose 20 mM Tris-HCl pH 6.8). Oocyte–cumulus complexes were recovered from the follicles that were 3–8 mm in size; the oocytes were separated individually from their cumulus cells (CC), and then rinsed with Tris-Sucrose buffer and snap-frozen in liquid nitrogen. Samples were kept at −80 °C and analyzed within no longer than 4 days.

For transcriptomic analysis, pools of GC, TH, CC, and 50 oocytes (*n* = 4 each) were collected and kept in TriZol reagent (Invitrogen, Cergy Pontoise, France) at −80 °C for RNA extraction.

### 4.4. Lipid Analysis 

#### 4.4.1. Nile Red Staining

Nile red staining of neutral lipids in bovine ovary sections was adapted from original protocol [98]. Bovine ovaries (*n* = 7) were frozen in nitrogen steam and cut at 10-μm slices using cryostat (MICROM NX70, Walldorf, Germany). Frozen sections were mounted onto Superfrost ^®^Plus slides (Thermo Scientific, Villebon sur Yvette, France) and air-dried for 10 min. Slides were fixed in 4% (*v*/*v*) of paraformaldehyde for 2 min, and then washed in water for 5 min at room temperature. The slides were incubated in 500 ng/µL of Nile red (NR) (Sigma Aldrich, St Louis, MO, USA) water solution for 1 h at room temperature, and then washed for 5 min in water and mounted in a Moveol solution complemented with 1 µg/mL of 4′,6-Diamidine-2′-phenylindole dihydrochloride (DAPI). Sequentially, NR slides were stained by hematoxylin-eosin for the precise identification of histological structures.

NR-treated slides were scanned using an AxioScan.Z1 scanner (Carl Zeiss SAS, Oberkochen, German) at 20×/0.8 magnification and using specific filters to detect blue DAPI fluorescence (excitation and emission wavelength were 335–383 nm and 420–470 nm, respectively) and NR fluorescence (excitation 538–562 nm and emission 570–640 nm) as described. The same exposure time were used for all of the slides (50 ms to DAPI and 35 ms to NR). Each image was analyzed using Zen Blue version 6.1.7601 software (Carl Zeiss Microscopy, GmbH, German).

In each image, the diameters of the follicles were measured. For each antral follicle, the intensity of NR fluorescence was measured in 25 regions of interest (ROI) positioned in FF, theca (TH), and granulosa cell (GC) layers, representing about 2 mm² ROI per compartment. The background was subtracted from the NR fluorescence intensity value, and the resulted value was normalized by the ROI exact area. A comparison of the normalized NR intensities between FF, GC, and TH was performed by one-way ANOVA followed by a Newman-Keuls multiple comparison test using GraphPad Prism version 5.01 (GraphPad Software, La Jolla, CA, USA).

#### 4.4.2. Lipid Identification Using Liquid Chromatography Coupled to High-Resolution Mass Spectrometry (LC-HRMS)

Three samples of 50 µL of FF or 10 mg of ovarian follicular cells were extracted according to a modified Folch method [99]. Briefly, samples were extracted with chloroform/methanol 2:1 (*v*/*v*), and 10 µL of internal standard mixture were added. Samples were then reconstituted with 100-fold dilutions of initial volume (50 µL for FF and 100 µL for cells) of MeOH/IPA/H_2_O 65:35:5 (*v*/*v*/*v*), vortexed for 30 s, and sonicated for 60 s before injection. Samples from follicular cells and FF were analyzed in two separated batches.

Total lipid extracts were separated on HTC PAL-system (CTC Analytics AG, Zwingen, Switzerland) coupled with a Transcend 1250 liquid chromatographic system (ThermoFisher Scientific, Les Ulis, France) using a kinetex C8 2.6 µm 2.1 × 150 mm column (Phenomenex, Sydney, NSW, Australia). Mobile phase A consisted of H_2_O/MeOH 60/40 (*v*/*v*) and 0.1 % formic acid and mobile phase B of IPA/MeOH 90/10 (*v*/*v*) and 0.1 % formic acid. Ammonium formate (10 mM) was added to both mobile phases in the positive ion mode. The gradient program from 32% to 95% of solvent B was established. The flow rate was 400 µL/min, and the column temperature was set to 60 °C.

Mass spectrometry analysis were performed using 10 µL of each sample. After injection, the column effluent was directly introduced into the heated electrospray (HESI) source of a Q-Exactive mass spectrometer (Thermo Scientific, San Jose, CA, USA), and analysis was performed in both ionization modes in two separate runs. Spray voltage was set to 3.7 kV and −3.1 kV in the positive and negative ionization modes, respectively. Mass spectra were recorded in full-scan MS mode from *m*/*z* 50 to *m*/*z* 2000 at a targeted mass resolution of 140 k, full width at half-maximum at *m*/*z* 200, and at a scan speed of 2 Hz.

Data were process as described previously [47]. Peak detection, correction, alignment, and integration were processed using XCMS R package with CentWave algorithm [49]. Features were then annotated thanks to an in silico lipid database and based on accurate measured masses, retention time windows, and relative isotopic abundance (RIA) of lipid species. A comparison of lipid families and lipid classes distributions was performed using Chi-2 following by Fisher exact test. Differences were considered significant at *p* < 0.05.

#### 4.4.3. Lipid Identification Using Tandem High-Resolution Mass Spectrometry (HRMS/MS)

Total lipids were extracted from 100 µL of follicular and environ 100 mg of follicular cells suspension as described [99,100].

From 10 μm, cryosections of freshly frozen bovine ovaries, in situ lipid extraction experiments were performed manually at multiple user-defined points across the tissue targeting follicles. For LESA, 1.5 μL of solvent were dispensed onto the tissue surface using a pipette, incubated for 5 s and reaspirated. Extractions were repeated four times in the same zone, and pooled, using two different extraction solutions as chloroform/methanol/2-isopropanol (1:2:4 *v*/*v*/*v*) or methanol 100%.

In order to obtain structural information for fine identifications, all of the lipid extracts were analyzed by tandem high-resolution mass spectrometry (HRMS/MS). All of the samples were infused directly in a dual linear ion trap Fourier Transform Mass Spectrometer (FT-MS) LTQ Orbitrap Velos (Thermo Fisher Scientific, Bremen, Germany). Data acquisitions were performed with Xcalibur software (version 2.1, Thermo Fisher Scientific, Bremen, Germany). FT-MS spectra were acquired using the profile mode in the 400–2000 *m*/*z* mass range, and the FT-MS/MS spectrum using HCD (high-energy collisional dissociation between 25–33%) fragmentation ion mode. Target resolution was set at 100,000 for FT-MS and FT-MS/MS analysis. The selected precursor width for fragmentation was 1.5 *m*/*z*.

Folch and LESA extracts were diluted (1:1, *v*:*v*) with a solution of 50% methanol/1% formic acid and loaded into a metalized nanoelectrospray needle (PicoTip emitters, New Objective) with a spray voltage of 1.2 kV. Spectra were acquire for 10 min in the positive ion mode from 400 *m*/*z* to 2000 *m*/*z*, using a method excluding automatically the precursors previously selected for the fragmentation using HCD collision energy of 25% and 33%.

Bligh & Dyer dried extracts were re-solubilized in a solution of 80% methanol/20% H_2_O in the presence of 1% formic acid and in a solution of 80% methanol/20% ammonium acetate of 100 mM for MS acquisitions in the positive and negative ion modes, respectively. FT-MS and FT-MS/MS spectra were acquired, over 10 min, in triplicate, using different collision energy levels (20 keV to 60 keV with a step of 5 keV). Data were collected using spectral stitching technique [101] as a series of 100-*m*/*z* wide windows that overlap by 5 *m*/*z*. Peaks were considered when >500 counts.

The data from FT-MS and FT-MS/MS were converted to Mascot generic format (MGF) using Proteome Discoverer software (version 1.4, ThermoFisher Scientific, San Jose, CA, USA) or MS convert integrated in an R-based tool as LipidMatch software [102]. All of the automatic lipid identifications that were proposed by LipidMatch (mass accuracy of 0.05 Da for precursor and 50 ppm for fragment ions, score = level 1) were confirmed by manual interpretation using LipidBlast 2.0 software. All of the identifications with a score Rev-Dot > 800 were accepted considering the presence of specific fragments ions (error mass accuracy of 0.8 Da) for phospholipids and sphingolipids. For each of the identified lipids, the theoretical mass and formula were obtained through interrogation of the LipidMaps database (http://www.lipidmaps.org/tools/ms/LMSD_search_mass_options.php).

#### 4.4.4. MSI (Mass Spectrometry Imaging) by MALDI-TOF MS

Freshly frozen whole ovaries were cut using a Cryo-Star HM 560 cryostat (Microm, Francheville, France) at −18 °C. The 10-µm thick sections were thaw-mounted onto conductive Indium Tin Oxide-coated microscope slides (Bruker Daltonics, Wissembourg, France). For external mass calibration, 1 μL of a mixture of small molecules and peptides (1 µL of calibrant solution containing Cafein, MRFA peptide, Leu-Enkephalin, Bradykinine 2–9, Glu1-fibrinopeptide B; reserpine; Bradykinine; Angiotensine I) was placed near the tissue section and mixed (1:1 *v*/*v*) with the matrix employed for the MALDI MSI.

Ovary sections were scanned before matrix deposition using a histology slide scanner (OpticLab H850 scanner, Plustek, Ahrensburg, Germany). These images were used to overlay the histological and molecular images. Slides were coated with a CHCA (α-cyano-4-hydroxycinnamic acid) matrix sprayed at 7 mg/mL in 60/40 acetonitrile/H_2_O, 0.2% trifluoroacetic acid using an Image Prep device (Bruker Daltonik GmbH, Bremen, Germany), and then vacuum-dried. Spectra were acquired using an UltrafleXtreme MALDI-TOF instrument (Bruker Daltonik GmbH, Bremen, Germany) equipped with a Smartbeam laser (Nd:YAG, 355 nm) monitored by the FlexControl 3.4 software (Bruker Daltonics, Bremen, Germany). MSI sequence was performed from one ovarian section targeting several regions of interest (ROIs) corresponding to individual antral follicles (2–6 mm in diameter).

Spectra were acquired in the positive mode, at a 2.0-kHz laser repetition rate, in the 100–1200 *m*/*z* range, using a spatial resolution set at 25 µm (medium focus setting) and collecting 500 spectra per pixel as a sum of 50 consecutive laser shots in 10 random walk shot steps. For a better mass accuracy across a tissue section image, raw spectra from all of the datasets were first aligned to a lock mass set at *m*/*z* 760.5780 corresponding to [M + H]^+^ of 34:1 PC. The unprocessed MSI sequence was then imported into the SCiLS Lab software (version 2016b, SCiLS, GmbH, Bremen, Germany). MSI sequence was loaded and pre-processed, according the used instrument type (TOF). Data were baseline reduced using a convolution algorithm with the setting of 20 for peak width and normalized to the total ion count (TIC). For each ROI (individual follicle), two-dimensional (2D) ion density maps using medium denoising were created from the average projection spectrum, and after data partitioning (bisecting k-Means), a segmentation map was generated.

#### 4.4.5. MALDI-TOF MS Analysis of Lipids on Follicular Cells and Fluid

Frozen oocytes were thawed in ice and placed on the MTP Ground Steel 384 MALDI plate (Bruker Daltonics, Germany) to dry. TH, GC, and CC samples were defrosted in an ice bath, and 5 µl of cell suspension or FF were dried by SpeedVac System (SPD 1010-230, Thermo Savant Eletronic Corporation, France) for 10 min at 45 °C. Pellets were re-suspended in a DHAP (2,5-dihydroxyacetophenone) matrix at 20 mg/mL solubilized in 90% methanol, 2% trifluoroacetic acid in water (4 µl for cells and 8 µL for FF samples). The matrix/sample mixtures were sonicated for four min using an ultrasonic bath sonicator (FisherBrand 15,052, Fisher Scientific) to solubilize the lipids. One microlitrer of the matrix/sample mix was overlaid with 1.5 µL of the same solution as the matrix. Each sample was spotted onto a MALDI plate in three replicates (with the exception of a single oocyte), and then dried at room temperature for 30 min. 3000 spectra per spot (as a sum of 1000 consecutive laser shots in three shot steps) were acquired, using a Bruker UltrafleXtreme MALDI-TOF instrument (Bruker Daltonics, Germany). Spectra were acquired in the positive and negative ion modes using reflecton in the *m*/*z* 100–1200 range. External calibration was performed using a mixture of small molecules and peptides (1 µL of calibrant solution containing Cafein, MRFA peptide, Leu-Enkephalin, Bradykinine 2–9, Glu1-fibrinopeptide B; reserpine; Bradykinine; Angiotensine I) and 1 µL of the DHAP matrix. To increase the mass accuracy (mass error < 0.05%), internal calibration was subsequently applied to all of the spectra. Lock mass correction was performed with highest intensity peak, corresponding to the glycerophosphatidylcholine (PC 32:1, *m*/*z* 732.556) in the positive mode, and glycerophosphatidylinositol (PI 38:4; *m*/*z* 885.5499) in the negative mode, using FlexAnalysis 4.1 software (Bruker). Each spectrum was converted to a .txt file using FlexAnalysis 3.4 software and processed using MALDI Progenesis software version 1.2 (Nonlinear Dynamics, Newcastle upon Tyne, UK). Peaks were automatically detected. The peaks with a signal to background ratio of five, and the isotopes were not considered for analysis. The coefficient of variation (CV%) of intensity values (normalized peak height, NPH) was calculated for all the peaks in three technical replicates per sample, from 12 different follicles for each tissue (Table A2, Appendix A). NPH values were used for a comparative analysis of lipid profiling between the compartments. One-way (ANOVA) and Tukey post-hoc test were used for differential analysis using XLSTAT software (Addinsoft, Paris, France). Differences were considered significant at *p* ≤ 0.05.

### 4.5. Gene Expression Analysis 

#### 4.5.1. RNA Extraction

Total RNA was extracted from the pools of isolated TH (*n* = 4), GC (*n* = 4), CC (*n* = 4) and oocytes (four pools of 50 oocytes) using TriZol reagent (Invitrogen, Cergy Pontoise, France) and then treated by RQ1 DNAse (Promega, Charbonnières, France) following the manufacturer’s instructions. After isopropanol precipitation, the RNA concentration was determined using a NanoDrop ND-1000 spectrophotometer (Nyxor Biotech, Paris, France), RNA integrity was checked on a Bioanalyzer 2100 (Agilent Technologies, Santa Clara, CA, USA).

#### 4.5.2. Microarray Hybridization and Transcriptome Data Analysist

A customized 60-K bovine microarray (Agilent Technologies, Santa Clara, CA, USA) covering more than 97% of the Ensembl Bos taurus transcripts (genome assembly UMD3.1) was used (GEO number GPL21724). Cyanine-3 (Cy3) labeled cRNA were prepared with 200 ng of total RNA using the One-Color Low Input Quick Amp Labeling kit following the recommended protocol (Agilent Technologies). Specific activities and cRNA yields were determined using the NanoDrop ND-1000. For each sample, hybridization was performed using 600 ng of Cy3-labeled cRNA (specific activity > 6.0 pmol of Cy3/μg of cRNA) for 17 h at 65 °C, following the manufacturer’s protocol at the Animal Biological Resources for Integrative and Digital Genomics facility (INRA Jouy-en-Josas, France, http://crb-gadie.inra.fr/). Slides were washed, air-dried, and scanned immediately using a G2565CA Scanner System with one-color scan setting (Agilent Technologies). The resulting images were analyzed using Feature Extraction Software v10.7.3.1 (Agilent Technologies). Raw data were normalized through intra-array median subtraction and log2 transformation, and then, the expression values were analyzed with a mixture model variance (VarMixt) method (R software). Raw probability values were adjusted using the Benjamini–Hochberg corrections for all of the comparisons; a differential was considered significant at *p* < 0.05. The gene ontology analysis of differential genes was performed using Enrichr gene set enrichment analysis web server application http://amp.pharm.mssm.edu/Enrichr/ [103]. The analysis of differential expression of lipid metabolism-related genes between follicular cells (TH, GC, CC a, d OO) was performed on the normalized expression values of 468 selected genes involved in lipid metabolism by applying ANOVA with Benjamini–Hochberg post-hoc correction of *p*-values. Multiple pair analysis was performed by Tukey test.

#### 4.5.3. Real Time PCR Expression Analysis

Total RNA was extracted as described in Section 4.5.1, from four independent pools of TH, GC, CC, and oocytes (*n* = 50 per pool). Reverse transcription (RT) was performed on 200 ng of extracted RNA from somatic cells and a total yield of oocyte RNA, using the Maxima First Strand cDNA Synthesis kit (Thermo-Fisher Scientific, Courtaboeuf, France) according to the manufacturer’s instructions. Real-time PCR reactions were carried out on a CFX96 (Bio-Rad, Marnes-la-Coquette, France) in 20 μL containing primers at a final concentration of 150 nM each, 5 μL of the diluted RT reaction (equivalent of 2 ng cDNA), and qPCR Mastermix Plus for SYBR Green I (Bio-Rad, Marnes-la-Coquette, France) according to the manufacturer’s instructions. The efficiency of the primers (Table A3, Appendix A), and standard curve for each gene was deduced from serial dilutions of the correspondent cDNA fragment obtained as a template. The geometric mean of three housekeeping genes (RPL19, RPS9, and GAPDH) was used to normalize gene expression. The relative expression of the genes of interest (RE) in each sample (*n* = six per cell type) was calculated according to the equation: *R* = gene E^-Ct^ gene/geometric mean (RPS9^E-Ct RPS9^; RPL19^E-Ct RPL19^; GAPDH^E-Ct GAPDH^), where *E* is the primer efficiency and Ct the cycle threshold.

Statistics were done using ANOVA followed Tukey post-hoc test. Difference was considered significant at *p* < 0.05.

## 5. Conclusions

Detailed data on lipid identification and their localization inside of the bovine ovarian follicle coupled with the tissue-specific expression of genes related to lipid turnover, suggest the potential capacity of follicular cells to transform FA from the nutritive uptake and use them to supplement the oocyte and follicular cells with membrane lipids, energy, and signaling mediators, thus maintaining follicular homeostasis. Data on the lipidomics and transcriptomics of ovarian follicular compartments that have been reported here might be useful for better understanding of the mechanisms of lipid metabolism in the oocyte and surrounding follicular cells.

## Figures and Tables

**Table 1:** Classification and distribution of lipids detected in bovine ovarian follicle.

**Lipid Family/**% from total lipids	**Lipid Classes/**% from total lipids
**Fatty acyls (FA)/**1.48%	Free fatty acid (**FFA**)/0.91%; Fatty acyl carnitine (**FAC**)/0.57%.
**Glycerolipids (GL)/**28.7%	Diacylglycerol (**DG)**/1.48%; Triacylglycerol (**TG**)/27.2%.
**Glycerophospholipids (GPL)/**52.56%	Lyso-phosphatidylcholine (**LPC**)/3.64%, phosphatidylcholine (**PC**)/30.33%; Lyso-phosphatidylethanolamine (**LPE**)/0.22%; phosphatidylethanolamine (**PE**)/12.88%; Lyso-phosphatidylinositol (**LPI**)/0.11%, phosphatidylinositol (**PI**)/1.71%; phosphoserine (**PS**)/2.85%; Glycerophosphates **(**here phosphatidic acids, **PA**)/0.23%; Phosphatidylglycerols (**PG**)/0.46%.
**Sphingolipids (SL)/**9.46%	Ceramides (**Cer**)/1.25%; Sphingomyelin (**SM**)/6.50%; Hexosylceramide (**HexCer**)/0.29%, Ganglioside (**GM**)/0.45%; Sulfoglycosphingolipid (**SuSM**)/1.02%.
**Sterol Lipids (ST)/**7.75%	Cholesteryl ester (**CE**)/7.41%; Sterols (**ST**)/0.34%

**Table 2:** Cluster composition of differentially expressed genes.

Clusters	*Differentially Expressed Genes*
Cluster 1 **(TH, GC)**	*ABHD12*, *ABHD13*, *ACADM*, *ACSBG1*, *ACSS1*, *AKR1E2*, *BDH2*, *CLN1*, *CYP2C87*, *CYP4V2*, *CYP7A1*, *ESR2*, *GJA1*, *GPAM*, *GPD1*, *GPX1*, *GPX3*, *GPX7*, *GPX8*, *HMGCL*, *HSDL1*, *HSDL2*, *LIPA*, *MCCC1*, *MLYCD*, *OSBPL7*, *PLA2G16*, *PLA2G1B*, *PLBD2*, *PLSCR2*, *SPTSSA*, *SULT2A1*, *UGCG*
Cluster 2 **(TH, GC, CC)**	*ABHD14B*, *ABHD3*, *ABHD7*, *ACAD10*, *APOA2*, *APOC3*, *APOE*, *APOL3*, *APOM*, *CD36*, *ACSS3*, *CYP4F2*, *DAGLB*, *HSD17B11*, *HSD17B7*, *LCAT*, *MCCC2*, *OSBPL1A*, *PIK3C2G*, *PLCG1*, *PLSCR1*, *PNPLA5*, *PNPLA7*, *PPARGC1A*, *SCARB2*, *SCP2*, *SCD5*
**Enriched GO terms** (clusters 1–2): Ketone body, phospholipid scrambling, cholesterol binding, sterol transporter activity, phospholipase activity, acyl-CoA, glycerophospholipid biosynthetic processes, FA oxidation, unsaturated FA metabolism, cellular response to oxidative stress. Pathways: arachidonic acid metabolism, PPAR and AMPK signaling, glutathione and glycerophospholipid metabolism.	
Cluster 3 **(CC)**	*ABHD15*, *ABHD16A*, *ACADL*, *ACADS*, *ACADVL*, *ACAT1*, *ACOX2*, *AGPAT5*, *AKR7A2*, *CDS2*, *CHPT1*, *CHST14*, *COMTD1*, *CYP19A1*, *CYP20A1*, *CYP51A1*, *DECR1*, *DGKA*, *FADS2*, *G6PC3*, *GPAA1*, *HSD11B1L*, *HSD3B*, *HSD3B7*, *LDLR*, *PI4KA*, *PIP5K1A*, *PKM2*, *PLCB4*, *PLD3*, *PPAP2A*, *SDHA*, *SLC27A3*, *SLC2A1*, *SLC2A3*, *SMPD1*, *SRD5A1*, *STARD7*, *STARD3NL*, *SUCLG1*
Cluster 5 **(CC)**	*CHST11*, *CHST8*, *CYP2U1*, *DGKE*, *HSD17B1*, *LSS*, *OSBPL2*, *PFKFB3*, *PLA2G7*, *PNPLA6*, *PPARG*, *SCD*, *UGGT2*
**Enriched GO terms** (clusters 3, 5): FA beta-oxidation using acyl-CoA dehydrogenase, steroid, organic cyclic compound, glycerophospholipid biosynthetic processes; FA beta-oxidation, FA catabolic process, estrogen biosynthesis, bile acid metabolic process, regulation of cholesterol metabolic process. Pathways: glycerophospholipid and FA metabolism, phosphatidylinositol and phospholipase D signaling; PPAR, choline metabolism, FA degradation, steroidogenesis, AMPK signaling	
Cluster 4 **(TH)**	*ACSF2*, *AGMO*, *AGPAT4*, *APOA1*, *CH25H*, *CHST1*, *CHST7*, *CYP27A1*, *CYP7B1*, *LPL*, *OXCT1*, *PDK4*, *PFKM*, *PIK3CG*, *PIK3R2*, *PLCD3*, *PLCXD3*, *PLD4*, *PLIN5*, *PLSCR4*, *PLTP*, *PPAP2A*, *PTPLA*, *SCARB1*, *SDHD*, *SMPD3*, *SMPDL3B*, *STARD5*, *SULT1A1*, *TM7SF2*
**Enriched GO terms** (cluster 4): Sterol import, regulation of cholesterol storage, regulation of sequestering of triglyceride and triglyceride synthesis; monocarboxylic acid bile acid biosynthetic process; sterol metabolic process. Pathways: PPAR signaling, primary bile acid biosynthesis, glycerolipid metabolism; Phospholipase D signaling pathway, Phosphatidylinositol signaling system, HDL-mediated transport	
Cluster 6 **(CC, OO)**	*ABHD11*, *ACADSB*, *ACSL6*, *AKR1A1*, *C2CD2L*, *CHST10*, *OGFOD2*, *OSBP2*, *PDK1*, *PPRC1*, *PPT2*, *SLC2A8*, *SMPD4*, *SOAT1*, *THEM4*, *UGT*, *MCC*
Cluster 7 **(OO)**	*ABHD10*, *ABHD17C*, *ABHD4*, *ABHD5*, *ABHD6*, *ACAA1*, *ACACA*, *ACAD11*, *ACAT2*, *ACER3*, *ACO1*, *ACOT7*, *ACOT9*, *ACP6*, *ACSL3*, *ACSL4*, *ACYP1*, *AGPAT9*, *AKR1B1*, *APOO*, *C2CD4A*, *C2CD5*, *CDS1*, *CEPT1*, *CERKL*, *CERS2*, *CHERP*, *CHKA*, *CHST12*, *CPT1C*, *CRLS1*, *CS*, *CYP26A1*, *CYP27B1*, *DECR2*, *DGAT2*, *DGKI*, *EBPL*, *ELOVL3*, *ELOVL4*, *ELOVL5*, *ELOVL7*, *EPT1*, *FABP3*, *FABP5*, *FADS3*, *FAR1*, *GK*, *GPAT2*, *GPD1L*, *GPLD1*, *HADHB*, *HMGCS1*, *HSD11B2*, *HSD17B12*, *LPCAT2*, *MBOAT2*, *NFKBIE*, *OGFOD1*, *OSBPL10*, *OSBPL11*, *OSBPL3*, *OSBPL8*, *OSBPL9*, *OXSM*, *PDK2*, *PDK3*, *PFKFB1*, *PFKFB2*, *PI4K2B*, *PI4KB*, *PIK3C2A*, *PIK3CA*, *PIK3C3*, *PIK3CB*, *PIK3R1*, *PIP4K2A*, *PIP4K2B*, *PIP4K2C*, *PIP5K1B*, *PIP5K1C*, *PITPNA*, *PITPNM1*, *PLA2G12A*, *PLA2G15*, *PLAA*, *PLCG2*, *PLCH1*, *PLD6*, *PLIN2*, *PNPLA4*, *PPAP2C*, *PPARGC1B*, *PTPLAD2*, *SOD1*, *SOD2*, *SPTLC1*, *SPTLC2*, *SRBD1*, *STARD4*, *SUCLA2*, *SUCLG2*, *THEM2*, *TM7SF3*, *UGDH*, *UGP2*, *UGT8*
**Enriched GO terms** (clusters 6–7): Glycerophospholipid and phosphatidylinositol biosynthetic processes; long-chain fatty-acyl-CoA biosynthesis and acyl-CoA metabolic process; FA elongation (saturated and unsaturated) FA; lipid phosphorylation; sphingolipid metabolic process; membrane lipid biosynthetic process. Pathways: Phosphatidylinositol and inositol lipid-mediated signaling; PPAR signaling; FA elongation, FA degradation; GL and GPL metabolism; peroxisome, choline metabolism; sphingolipid metabolism	

## Supplementary Materials

Supplementary materials can be found at http://www.mdpi.com/1422-0067/19/10/3261/s1.

## Abbreviations

CC	Cumulus cells
Cer	Ceramids
CE	Cholesteryl esters
DG	Diacylglycerols
FA	Fatty acyls
FFA	Free fatty acids
FAC	Fatty acyl carnitines
FF	Folicular fluid
FSH	Follicle Stimulation Hormone
GC	Granulosa cells
GM	Ganglioside
GL	Glycerolipids
GO	Gene Onthology
GP	Glycerophosphates
GPL	Glycerophospholipids
HCer	Hexosylceramide
HDL	High Density Lipoprotein
Hcer	Hexosylceramides
LC/MS	Liquid Chromatography/Mass Spectrometry
LC-HRMS	Liquid Chromatography- High Resolution Mass Spectrometry
LD	Lipid droplet
LDL	Low Density Lipoprotein
LH	Luteinizing Hormone
LPA	Lyso-phatidic acid
LPC	Lyso-phosphatidylcholines
LPE	Lyso-phosphatidylethanolamines
LPI	Lyso-phosphatidylinositols
LPL	Lipoprotein Lipase
*m*/*z*	Ion Mass to Ion Charge number ratio
MALDI-TOF	Matrix Assisted Laser Desorption Ionization -Time of Flight
MS	Mass Spectrometry
MSI	Mass Spectrometry Imaging
NEB	Negative Energy Balance
NR	Nile Red
OO	Oocyte
OOC	Oocyte-cumulus complex
PA	Phosphatidic Acid
PC	Phosphatidylcholines
PE	Phosphatidylethanolamines
PG	Phosphatidylglycerols
PI	Phosphatidylinositols
PS	Phosphatidylserines
SL	Sphingolipids
SM	Sphingomyelins
ST	Sterols/Steroid Derivatives
SuSM	Sulfoglycosphingolipids
TG (TAG)	Triacylglycerols
TH	Theca cells

## Appendix A

**Table A1:** Identification of the lipids detected by mass spectrometry imaging (Figure 3B).

Observed *m*/*z*	Exact *m*/*z*	Delta/Da	Lipid Annotation	Ion
104.195	104.173	−0.022	Choline	[M + H]^+^
184.189	184.152	−0.037	Phosphocholine	[M + H]^+^
496.434	496.34	−0.0937	LPC 16a:0	[M + H]^+^
520.427	520.34	−0.0867	LPC 18a:2	[M + H]^+^
522.429	522.356	−0.073	LPC 18a:1	[M + H]^+^
524.458	524.372	−0.0864	LPC 18a:0	[M + H]^+^
534.379	534.296	−0.0834	LPC 16a:0	[M + K]^+^
544.417	544.3403	0.0767	LPC 20a:4	[M + H]^+^
560.398	560.3116	−0.0867	LPC 18a:1	[M + K]^+^
703.64	703.575	−0.0652	SM(d18:1/C16:0)	[M + H]^+^
706.592	706.539	−0.0533	PC 30a:0	[M + H]^+^
725.587	725.557	−0.0302	SM(d18:1/C16:0)	[M + Na]^+^
732.609	732.5543	−0.0547	PC 32a:1	[M + H]^+^
734.626	734.57	−0.056	PC 32a:0	[M + H]^+^
741.581	741.5881	0.0071	SM(d18:0/C17:0)	[M + Na]^+^
746.617	746.57	−0.047	PC 33a:1	[M + H]^+^
748.625	748.5856	−0.0394	PC 33a:0	[M + H]^+^
753.628	753.5881	−0.0399	SM(d18:1/C18:0)	[M + Na]^+^
754.581	754.5357	−0.0453	PC 32a:1	[M + Na]^+^
755.59	755.5469	−0.0431	SM(d18:1/C17:0)	[M + K]^+^
756.599	756.5514	−0.0476	PC 32a:0	[M + Na]^+^
758.621	758.56995	−0.0511	PC 34a:2	[M + H]^+^
760.617	760.5856	−0.0314	PC 34a:1	[M + H]^+^
768.598	768.5514	−0.0466	PC 33a:1	[M + Na]^+^
769.589	769.5626	−0.0264	SM(d18:1/C18:0)	[M + K]^+^
770.551	770.567	0.016	PC 33a:0	[M + Na]^+^
772.562	772.5253	0.0367	PC 32a:0	[M + K]^+^
774.634	774.6013	−0.0327	PE 38:1	[M + H]^+^
780.578	780.5514	−0.0266	PC 34a:2	[M + Na]^+^
782.603	782.567	−0.036	PC 34a:1	[M + Na]^+^
784.602	784.5827	−0.0193	PC 34a:0	[M + Na]^+^
786.632	786.6013	−0.0307	PC 36a:2	[M + H]^+^
788.664	788.6169	−0.0471	PC 36a:1	[M + H]^+^
794.605	794.567	−0.038	PC 35a:2	[M + Na]^+^
796.562	796.5827	0.0207	PC 35a:1	[M + Na]^+^
798.578	798.5983	0.0203	PC 35a:0	[M + Na]^+^
806.583	806.567	−0.016	PC 36a:3	[M + Na]^+^
808.613	808.5827	−0.0303	PC 36a:2	[M + Na]^+^
810.615	810.5983	−0.0167	PC 36a:1	[M + Na]^+^
812.592	812.614	0.022	PC 36:0	[M + Na]^+^
822.567	822.5843	0.0173	PC 36a:3	[M + K]^+^
824.587	824.5566	−0.0304	PC 36a:2	[M + K]^+^
826.581	826.572	0.0087	PC 36a:1	[M + K]^+^

Identifications of the lipids with the lowest delta value (exact *m*/*z* –observed *m*/*z*) are shown. All of the identifications are available in Supplementary Data (Table S2). Abbreviations: a—acyl; LPC—lyso-glycerophosphocholines; PC—glycerophosphocholines; SM—sphingomyelins. M—mass; Na—sodium ion; K—potassium.

**Table A2:** Coefficients of variation (%) of normalized peak heights quantification from MALDI-TOF lipid profiles.

Compartment	Positive Mode	Negative Mode
TH	15.9%	16.1%
GC	18.4%	15.8%
FF	18.0%	22.9%
CC	27.6%	21.7%
OO	32.9%	25.3%

**Table A3:** List of primers used for RT-qPCR analysis of gene expression in bovine ovarian cells.

Gene	Accession Number	Description	Primer’s Sequence (5′-3′)		Efficiency, % (E)
*ABHD6*	NM_001075196	Abhydrolase domain containing 6 (ABHD6)	Fw	ACCCCGAAGGAGATGAGTGA	92.6% (1.93)
			Rev	CTGGGAGTTGGCGATTGACT	
*AGPAT9*	NM_001192514	Acylglycerol-3-phosphate O-acyltransferase 9	Fw	AATGCCTCTCCCATCCGTTG	88.9% (1.89)
			Rev	TTATGCTGCACAGTCGGGAA	
*CPT1A*	FJ415874	Carnitine palmitoyltransferase 1A	Fw	TCCTGGTGGGCTACCAATTA	95.4% (1.95)
			Rev	TGCGTCTGTAAAGCAGGATG	
*GAPDH*	NM_001034034	Glyceraldehyde 3 phosphate dehydrogenase	Fw	TTCAACGGCACAGTCAAGG	100.3% (2.00)
			Rev	ACATACTCAGCACCAGCATCAC	
*DGAT2*	NM_205793.2	Diacylglycerol O-acyltransferase 2	Fw	GTGGCCTCGCTTTGCTTAAC	92.3% (1.92)
			Rev	GGGTTCGGGGAACTTCTGTT	
*FABP3*	NM_174313	Fatty acid binding protein 3	Fw	ATCGTGACGCTGGATGGCGG	104.1% (2.04)
			Rev	GCCGAGTCCAGGAGTAGCCCA	
*FASN*	AY343889	Fatty acid synthase	Fw	CACTCCATCCTCGCTCTCC	102.9% (2.02)
			Rev	GCCTGTCATCATCTGTCACC	
*LPL*	NM_001075120	Lipoprotein lipase	Fw	GGGTTTTGAGCAAGGGTACA	87.1% (1.87)
			Rev	GCCACAATGACCTTTCCAGT	
*PLIN2*	NM_173980	Perilipin 2	Fw	ACAACACACCCCTCAACTGG	97.5% (1.96)
			Rev	CTGCCTGCCTACTTCAGACC	
*PNPLA2*	NM_001046005	Patatin-like phospholipase domain containing 2	Fw	ATGGTGCCCTACACTCTGCC	87.6% (1.88)
			Rev	AGCTTCCTCTTGGCGCGTAT	
*PPARG*	Y12419	Peroxisome proliferator-activated receptor gamma	Fw	CCCTGGCAAAGCATTTGTAT	105.1% (2.05)
			Rev	ACTGACACCCCTGGAAGATG	
*RPL19*	BC102223	Ribosomal protein L19	Fw	AATCGCCAATGCCAACTC	98.2% (1.98)
			Rev	CCCTTTCGCTTACCTATACC	
*RPS9*	BC148016	Ribosomic protein S9	Fw	GGAGACCCTTCGAGAAGTCC	85.0% (1.85)
			Rev	GGGCATTACCTTCGAACAGA	
